# Supplementary material for: Communication speeds up but impairs the consensus decision in a dyadic colour estimation task
Source: R Soc Open Sci. 2020 Jul 22;7(7):191974. doi: 10.1098/rsos.191974 (PMC7428237; doi:10.1098/rsos.191974)
Supplement: Supplementary information for DCE_RSOS [file rsos191974supp2.docx]

**Supplementary Information for**

**Communication speeds up but impairs the consensus decision in a dyadic color estimation task**

Liutao Yu^#^, Chundi Wang^#^, Si Wu and Da-Hui Wang*

^#^ LT Yu and CD Wang contributed equally to this work.

*To whom correspondence should be addressed. E-mail: wangdh@bnu.edu.cn

**This file includes:**

Supplementary Methods

Supplementary Figure 1 ~ 6

Supplementary Table 1 ~ 2

**Supplementary Methods.** In this section, we first introduced the details of three models: the virtual consensus by random sampling model (VCRS), the weighted confidence sharing model (WCS), and the direct signal sharing model (DSS). Then, we applied simple probabilistic analysis to explain the mechanism underlying the observation that consensus without communication is better than consensus with communication in a statistical sense.

**Virtual consensus by random sampling model (VCRS)**

In this study, wo proposed a simple VCRS model to mimic the consensus formation in the without-communication condition. First, we separated the experimental data into subsets according to different hues (each row in Fig. s1a accordingly) and different stimulus differences $\Delta$s. Then, for each subset, we conducted the following random sampling method to build virtual consensuses.

For each round, we randomly sampled two reports from two participants' first-round report collections, and calculated the difference between them. If the difference between two samples was less than $1^{\circ}$, i.e. the sampled reports reached a consensus, then a trial ended; otherwise, the trial moved to the next round. A trial ended before eight rounds whenever two samples reached a consensus, or ended right after the eighth round regardless of reaching a consensus or not.

We simulated 200 trials for each condition. And all the VCRS data were analyzed using similar methods as for the experimental data.

**Weighted confidence sharing model (WCS)**

The WCS model was proposed by Bahrami et al. 2010 [1]. In their study, two participants first individually made a two-alternative forced perceptual decision about whether a contrast oddball was in the first or second interval. Then, ﻿the participants were free to verbally discuss their choices with each other as long as they wanted, before one of them reported their dyadic decision. The authors applied psychometric curves fitted by the cumulative Gaussian function $P\left( \Delta c \right)=H(\frac{\Delta c+b}{\sigma})$ to measure the quality of decisions, where $\Delta c$ was the contrast difference between the second and first presentations, $P\left( \Delta c \right)$ corresponded to the probability of saying that the second interval had the higher contrast, and $H\left( z \right)=\int_{-\infty}^{z} \frac{dt}{\sqrt{2\pi}}exp[-t^{2}/2]$. Thus, the sensitivity was defined as the maximum slope of the psychometric curve $s=\frac{1}{\sqrt{2\pi}\sigma}$, and a large slope indicated small variance and thus highly sensitive performance.

In the WCS model, the authors assumed that two participants communicated their confidence, i.e. z-score $\Delta c/\sigma$, during oral discussion. Thus, the probability correct for the dyad was given by $P_{dyad}^{WCS}\left( \Delta c \right)=\int_{\frac{x_{1}}{\sigma_{1}}+\frac{x_{2}}{\sigma_{2}}>0} \frac{dx_{1}dx_{2}}{2\pi\sigma_{1}\sigma_{2}}\exp[-\frac{\left( x_{1}-b_{1}-\Delta c \right)^{2}}{2\sigma_{1}^{2}}-\frac{\left( x_{2}-b_{2}-\Delta c \right)^{2}}{2\sigma_{2}^{2}}]$. After some calculation, they obtained $P_{dyad}^{WCS}\left( \Delta c \right)=H(\frac{\Delta c+b_{dyad}^{WCS}}{\sigma_{dyad}^{WCS}})$, where $b_{dyad}^{WCS}=\frac{\sigma_{2}b_{1}+\sigma_{1}b_{2}}{\sigma_{1}+\sigma_{2}}$ and $\sigma_{dyad}^{WCS}=\frac{\sqrt{2}\sigma_{1}\sigma_{2}}{\sigma_{1}+\sigma_{2}}$. Expressed in terms of sensitivities, these equations became $b_{dyad}^{WCS}=\frac{s_{1}b_{1}+s_{2}b_{2}}{s_{1}+s_{2}}$ and $s_{dyad}^{WCS}=\frac{s_{1}+s_{2}}{\sqrt{2}}$. For more details, please refer to Bahrami’s paper [1].

Although the WCS model is proposed for a two-alternative perceptual decision scenario, it can be applied to analyze our data. Because in our tasks, the decision quality is measured by a report error distribution which can be fitted by a Gaussian function. Thus, we also obtain the bias and sensitivity to evaluate performance. And we can apply the WCS model to integrate individuals’ biases and sensitivities to obtain predictions of cooperation effects.

**Direct signal sharing model (DSS)**

The DSS model was named by Bahrami et al. 2010 [1], but actually proposed by Ernst and Banks 2002 to account for multi-sensory integration phenomenon [2]. The problem they studied was how a person integrate visual-haptic percept when one looked at an object while touching it with their hand. The authors proposed a general principle which minimized variance in the final estimate, by using maximum-likelihood estimation to combine the visual and haptic inputs.

The authors assumed that the estimate of an environmental property like size, shape or position, by a sensory system can be represented by $\hat{b_{i}}=f_{i}(b)$, where $b$ is the physical property being estimated and $f$ is the operation by the nervous system. Each estimate $\hat{b_{i}}$ is corrupted by noises, which are independent and Gaussian with variance $\sigma_{i}^{2}$. If the Bayesian prior is uniform, then the maximum-likelihood estimate of the environment is given by $\hat{b}=\sum_{i} w_{i}\hat{b_{i}}$ with $w_{i}=\frac{1/\sigma_{i}^{2}}{\sum_{j} 1/\sigma_{j}^{2}}$. And the variance of the final estimate $\hat{b}$ is $\sigma^{2}=\frac{\sigma_{1}^{2}\sigma_{2}^{2}}{\sigma_{1}^{2}+\sigma_{2}^{2}}$. Expressed in terms of sensitivity $s=\frac{1}{\sqrt{2\pi}\sigma}$, Bahrami et al. obtained $b_{dyad}^{DSS}=\frac{s_{1}^{2}b_{1}+s_{2}^{2}b_{2}}{s_{1}^{2}+s_{2}^{2}}$ and $s_{dyad}^{DSS}=\sqrt{s_{1}^{2}+s_{2}^{2}}$ [1].

The model well explains human behaviors in a visual-haptic task [2]. Later studies including Bahrami’s work [1], tried to extended this multi-sensory ﻿Bayesian inferential framework as a cue integration framework for social interaction and social cognition [3]. Therefore, in this study we also apply this cue integration framework to integrate two individuals’ sensitivity to obtain predictions of bias and sensitivity after cooperation, which then can be compared to other cooperation results.

**Why consensus without communication is better than that with communication?**

To explain the observation that consensus without communication is better than consensus with communication in a statistical sense, we classify the estimates into good or poor ones. A good estimate is close to the stimulus presented, while a poor estimate is far away from the stimulus. The probability of a poor consensus can be described as $P\left( b \right)=\frac{p(b_{1}, b_{2})}{p\left( b_{1}, b_{2} \right)+p(g_{1}, g_{2})}$, where $p\left( b_{1}, b_{2} \right)$(or $p(g_{1}, g_{2})$) is the probability that two participants simultaneously make poor (or good) estimates. For consensus with communication, estimates of one participant are dependent on the estimates of the other participant. Using the law of total probability, we can obtain the probability of reaching a poor consensus with communication $p_{w}\left( b \right)=\frac{p(b_{1})}{p\left( b_{1} \right)+p(g_{1})\frac{p(g_{2}|g_{1})}{p(b_{2}|b_{1})}}$, where $p(g_{2}|g_{1})$ (or $p(b_{2}|b_{1})$) is the conditional probability that one participant makes a good (or poor) estimate given a good (or poor) estimate by the other participant. In our task, it is reasonable that a poor estimate far away from the stimulus has stronger influence on estimates of the other participant than a good estimate close to the stimulus, which implies $\frac{p(g_{2}|g_{1})}{p(g_{2})}\leq\frac{p(b_{2}|b_{1})}{p(b_{2})}$. Thus, we can further obtain the following relationship:

$$p_{w}\left( b \right)\geq\frac{p\left( b_{1} \right)}{p\left( b_{1} \right)+p\left( g_{1} \right)\frac{p\left( g_{2} \right)}{p\left( b_{2} \right)}}=\frac{p\left( b_{1} \right)p\left( b_{2} \right)}{p\left( b_{1} \right)p\left( b_{2} \right)+p\left( g_{1} \right)p\left( g_{2} \right)}=p_{w/o}(b),$$

where $p_{w/o}(b)$ is the probability that a dyad reach a poor consensus without communication. Therefore, the probability of reaching a poor consensus with communication is larger than that without communication, indicating that consensus without communication is better than consensus with communication in a statistical sense.

**References:**

1. Bahrami B, Olsen K, Latham P, Roepstorff A, Rees G, Frith C. 2010. Optimally interacting minds. *Science* 329, 1081–1085.

2. Ernst M, Banks M. 2002. Humans integrate visual and haptic information in a statistically optimal fashion. *Nature* 415, 429–433.

3. ﻿Zaki, J. 2013. Cue Integration: A Common Framework for Social Cognition and Physical Perception. *Perspectives on Psychological Science*, 8(3), 296–312.

**Supplementary Figure s1.** Examples of the distributions of participant’s estimates both the first round (top) and the last round (bottom) for both members when $\Delta=9^{\circ}$ (left: Participant1; right: Participant2) in “*Aggregation across dyads*”. The mean estimate of the first round for both participants are near 0, while those of the last round deviate from 0 due to communication. Moreover, the standard deviations of the estimate of the first round for both participants are obviously higher than those of the last round, indicating that cooperation narrows the distribution of estimates.


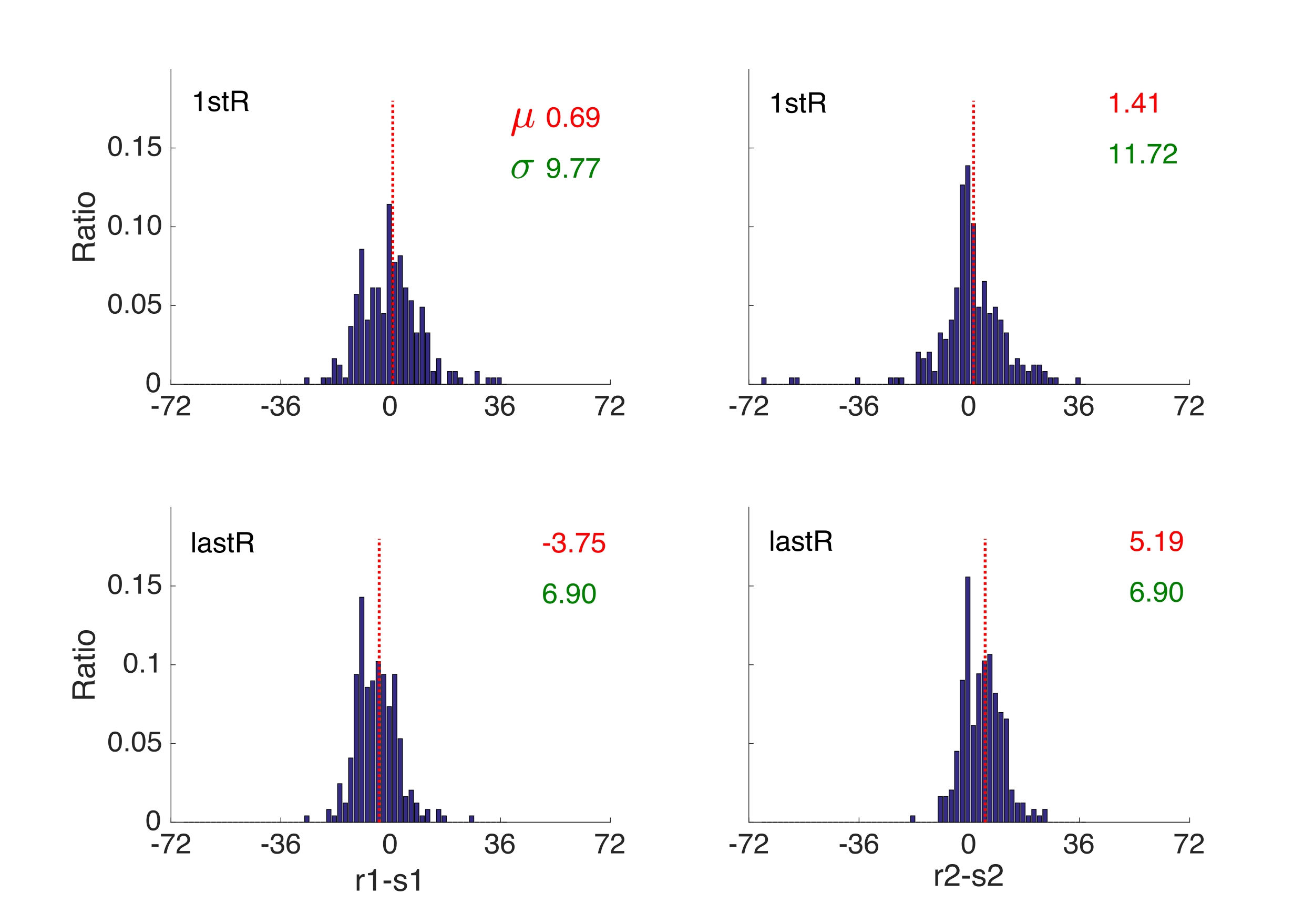


**Supplementary Figure s2.** In the without-communication condition, the performance (measured by the mean and standard error of means (s.e.m.) of the estimate distribution) of a participant does not show consistent improvement with the estimation proceeding, in trials with $\Delta=0^{\circ}$ and no consensus forms. This observation allows us to apply the first-round estimates as approximations for the participants' individual estimates, which are not influenced by communication.


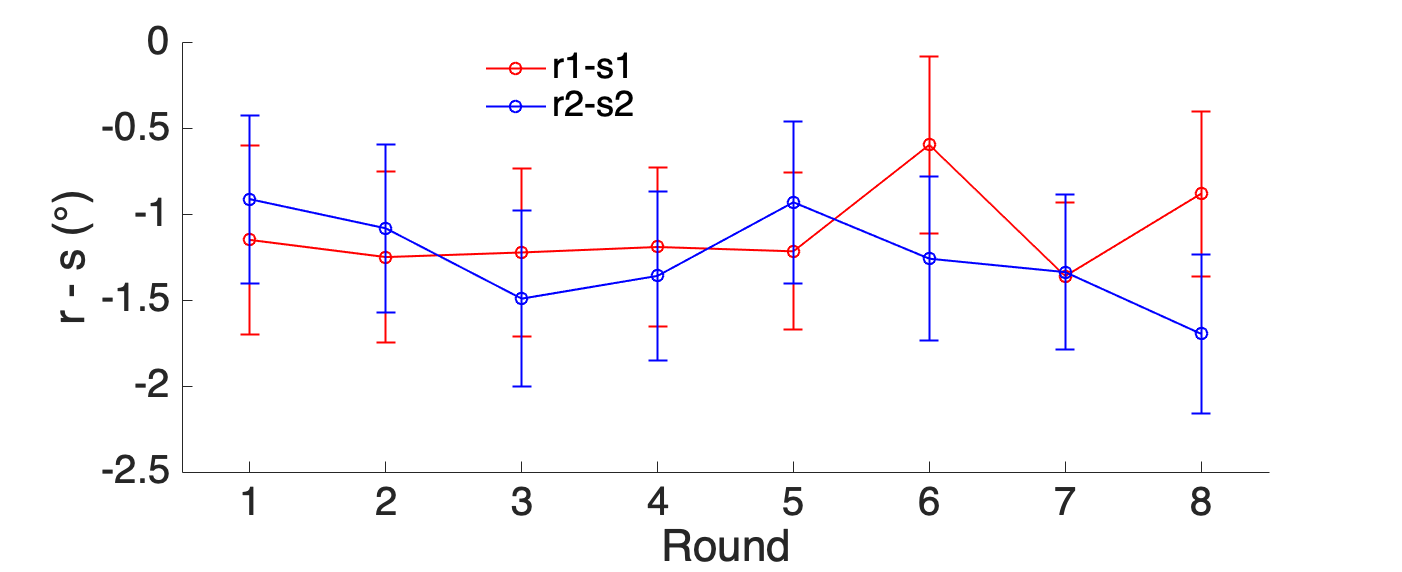


**Supplementary Figure s3.** The relationship of the mean estimates between two participants for both the first round (left column) and the last round (right column) are roughly independent of the absolute color of the stimuli, while they are mainly dependent on the stimulus difference $\Delta$, which is why we focus our analyses on the effect of $\Delta$. Error bars indicate s.e.m.


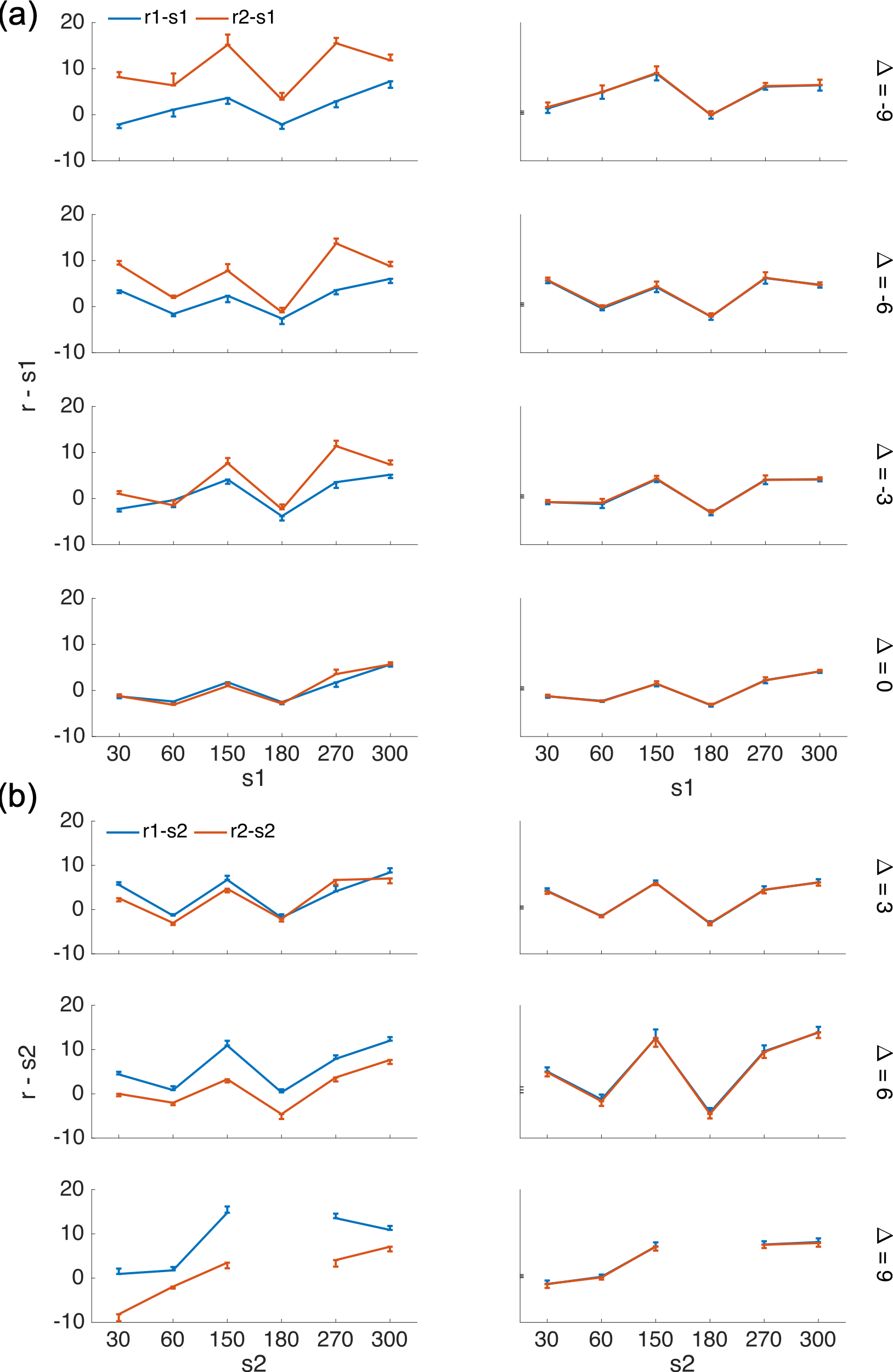


**Supplementary Figure s4.** Comparison of the mean estimates $<r-s>$ among single participants, with-communication dyads, without-communication dyads, and several models including VCRS, WCS and DSS when $\Delta=0^{\circ}$. Error bars indicate s.e.m. Statistical test results can be found in Supplementary Table 1.


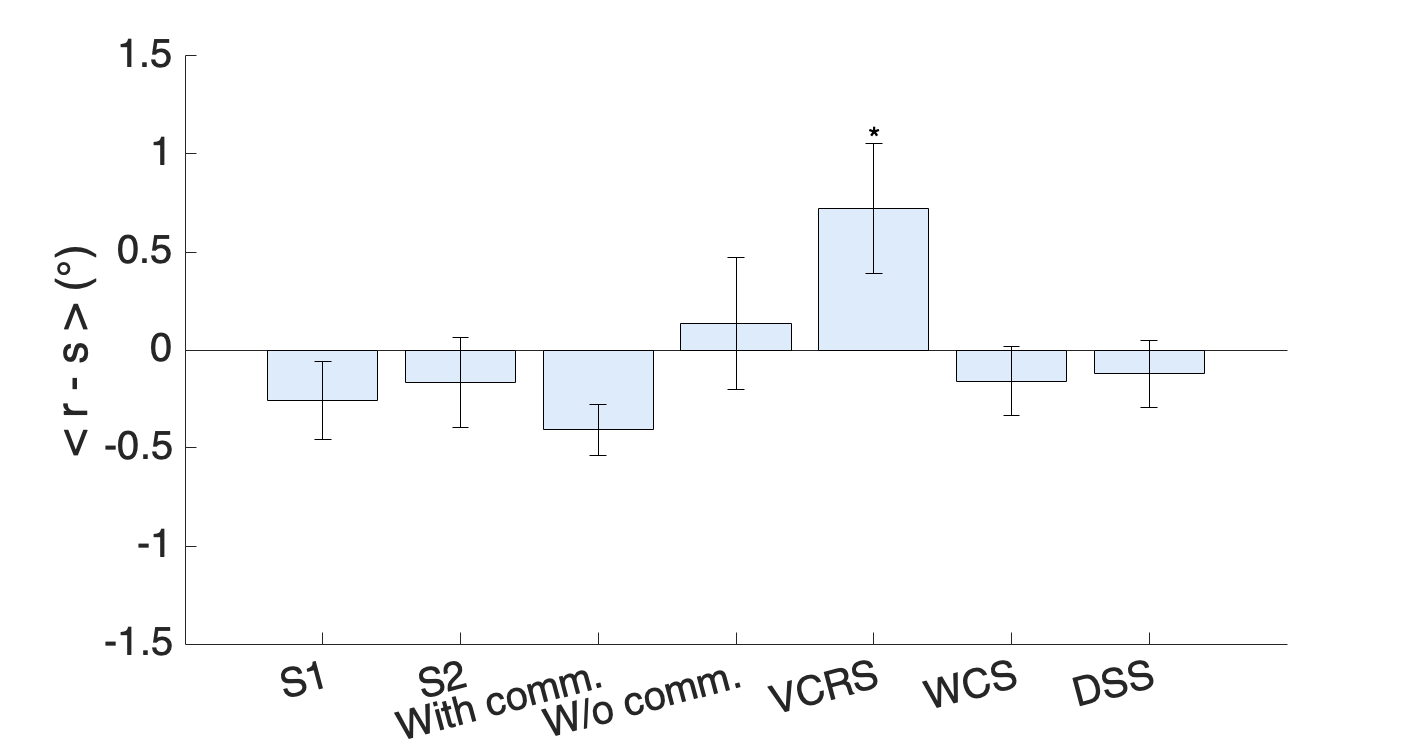


**Supplementary Figure 5.** The relationship between improvement ratio and ratio of intersections (RoI). Circles represent original data, while stars represent smoothed data.

**
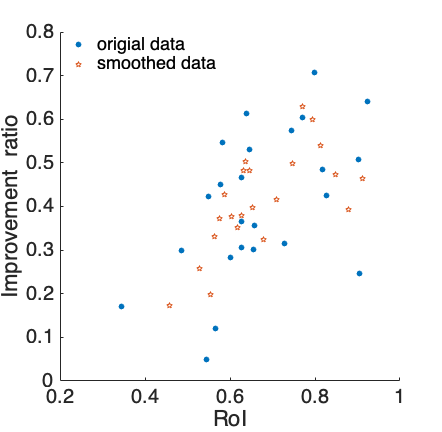
**

**Supplementary Figure s6.** Trial-end feedback has no significant effect on the improvement ratio of sensitivity (two-sample *t*-test: $h=0, t\left( 46 \right)=-0.26, p=0.79$). Error bars indicate s.e.m.


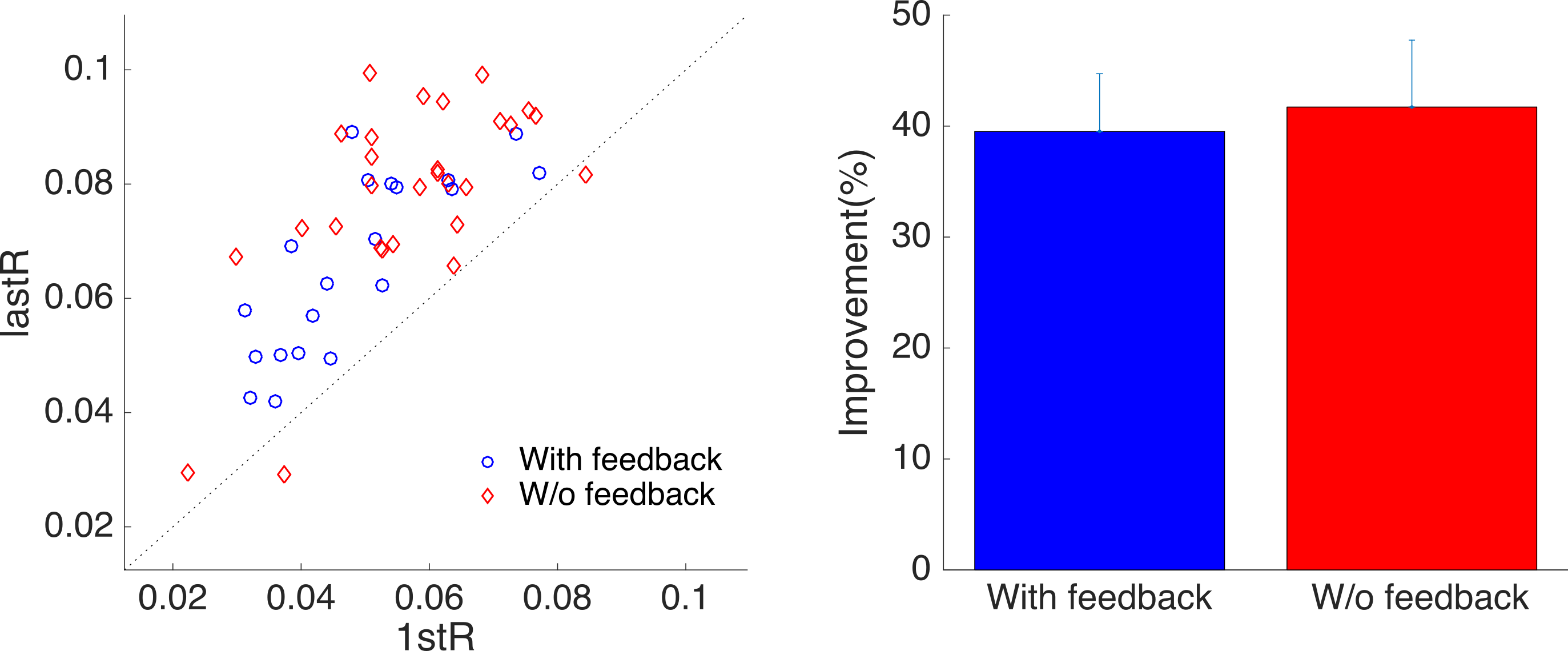


**Supplementary Table s1.** Statistical *t*-*test* (test for a mean equal to zero) results of $r-s$ when $\Delta=0^{\circ}$ (see Supp. Fig. 4). Multiple comparisons were corrected using Bonferroni correction (***, **, * mean $p<0.001, p<0.01, p<0.05$ respectively).

|  | P1 | P2 | With comm. | W/o comm. | VCRS | WCS | DSS |
| --- | --- | --- | --- | --- | --- | --- | --- |
| ***h*** | 0 | 0 | 0 | 0 | 1 | 0 | 0 |
| $\boldsymbol{t}_{\boldsymbol{stat}}$ | -1.286 | -0.722 | -2.206 | 0.404 | 3.064* | -0.909 | -0.704 |
| ***p*** | 1 | 1 | 0.263 | 1 | 0.025 | 1 | 1 |

**Supplementary Table s2.** Two-sample *t*-test results of sensitivities among different experimental conditions when $\Delta=0^{\circ}$ (see Fig. 4c in the *text*). Multiple comparisons were corrected using Bonferroni correction (***, **, * mean $p<0.001, p<0.01, p<0.05$ respectively).

| ***h*** | P1 | P2 | With comm. | W/o comm. |
| --- | --- | --- | --- | --- |
| P1 | - | 0 | 1 | 1 |
| P2 |  | - | 1 | 1 |
| With comm. |  |  | - | 1 |
| W/o comm. |  |  |  | - |

| $\boldsymbol{t}_{\boldsymbol{stat}}$ | P1 | P2 | With comm. | W/o comm. |
| --- | --- | --- | --- | --- |
| P1 | - | -0.816 | -5.829*** | -6.612*** |
| P2 |  | - | -4.667*** | -6.093*** |
| With comm. |  |  | - | -2.939* |
| W/o comm. |  |  |  | - |

| ***p*** | P1 | P2 | With comm. | W/o comm. |
| --- | --- | --- | --- | --- |
| P1 | - | 1 | 9.40E-07 | 1.93E-06 |
| P2 |  | - | 8.56E-05 | 5.96E-06 |
| With comm. |  |  | - | 0.034 |
| W/o comm. |  |  |  | - |

| ***h*** | With comm. | VCRS | WCS | DSS |
| --- | --- | --- | --- | --- |
| With comm. | - | 0 | 0 | 0 |
| VCRS |  | - | 0 | 0 |
| WCS |  |  | - | 0 |
| DSS |  |  |  | - |

| $\boldsymbol{t}_{\boldsymbol{stat}}$ | With comm. | VCRS | WCS | DSS |
| --- | --- | --- | --- | --- |
| With comm. | - | -2.0104 | 0.2846 | 0.0253 |
| VCRS |  | - | 2.4729 | 2.2207 |
| WCS |  |  | - | -0.3062 |
| DSS |  |  |  | - |

| ***p*** | With comm. | VCRS | WCS | DSS |
| --- | --- | --- | --- | --- |
| With comm. | - | 0.291 | 1 | 1 |
| VCRS |  | - | 0.092 | 0.174 |
| WCS |  |  | - | 1 |
| DSS |  |  |  | - |

|  | | VCRS | WCS | DSS |
| --- | --- | --- | --- | --- |
| W/o comm. | ***h*** | 0 | 1 | 1 |
|  | $\boldsymbol{t}_{\boldsymbol{stat}}$ | 1.513 | 3.263** | 3.089* |
|  | ***p*** | 0.404 | 0.008 | 0.012 |
